# Nanoemulsions and nanocapsules as carriers for the development of intranasal mRNA vaccines

**DOI:** 10.1007/s13346-024-01635-5

**Published:** 2024-05-29

**Authors:** Mireya L. Borrajo, Gustavo Lou, Shubaash Anthiya, Philipp Lapuhs, David Moreira Álvarez, Araceli Tobío, María Isabel Loza, Anxo Vidal, María José Alonso

**Affiliations:** 1 Center for Research in Molecular Medicine and Chronic Diseases (CiMUS), University de Santiago de Compostela, Av. Barcelona s/n, Campus Vida, Santiago de Compostela, 15782 Spain; 2 Department of Pharmacy and Pharmaceutical Technology, School of Pharmacy, University of Santiago de Compostela, Santiago de Compostela, 15782 Spain; 3 IDIS Research Institute, University of Santiago de Compostela, Santiago de Compostela, 15782 Spain; 4 Biofarma Research Group, Center for Research in Molecular Medicine and Chronic Diseases (CiMUS), University of Santiago de Compostela, Av. Barcelona s/n, Campus Vida, Santiago de Compostela, 15782 Spain

**Keywords:** mRNA vaccine, Nanoparticles, Polymeric nanocapsule, SARS-CoV-2, Intranasal vaccination

## Abstract

The global emergency of coronavirus disease 2019 (COVID-19) has spurred extensive worldwide efforts to develop vaccines for protection against severe acute respiratory syndrome coronavirus 2 (SARS-CoV-2). Our contribution to this global endeavor involved the development of a diverse library of nanocarriers, as alternatives to lipid nanoparticles (LNPs), including nanoemulsions (NEs) and nanocapsules (NCs), with the aim of protecting and delivering messenger ribonucleic acid (mRNA) for nasal vaccination purposes. A wide range of prototypes underwent rigorous screening through a series of in vitro and in vivo experiments, encompassing assessments of cellular transfection, cytotoxicity, and intramuscular administration of a model mRNA for protein translation. As a result, two promising candidates were identified for nasal administration. One of them was a NE incorporating a combination of an ionizable lipid (C12-200) and cationic lipid (DOTAP), both intended to condense mRNA, along with DOPE, which is known to facilitate endosomal escape. This NE exhibited a size of 120 nm and a highly positive surface charge (+ 50 mV). Another candidate was an NC formulation comprising the same components and endowed with a dextran sulfate shell. This formulation showed a size of 130 nm and a moderate negative surface charge (-16 mV). Upon intranasal administration of mRNA encoding for ovalbumin (mOVA) associated with optimized versions of the said NE and NCs, a robust antigen-specific CD8 + T cell response was observed. These findings underscore the potential of NEs and polymeric NCs in advancing mRNA vaccine development for combating infectious diseases.

**Graphical Abstract:**

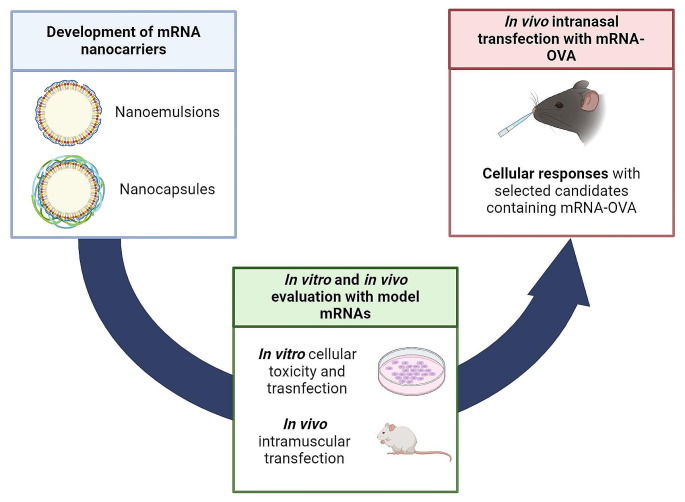

**Supplementary Information:**

The online version contains supplementary material available at 10.1007/s13346-024-01635-5.

## Introduction

On March 11, 2020, the World Health Organization (WHO) declared the coronavirus disease 2019 (COVID-19) outbreak as a global pandemic [1]. COVID-19 is caused by the highly contagious, pathogenic, and mutagenic severe acute respiratory syndrome coronavirus 2 (SARS-CoV-2), which has caused over 760 million confirmed cases, and nearly 7 million deaths worldwide [2–4]. In response to the urgent need to stop the spread of the virus and minimize the severity of COVID-19, research on messenger ribonucleic acid (mRNA) vaccines gained prominence [5]. Notably, the first two vaccines authorized by the European Medicines Agency (EMA) and the US Food and Drug Administration (FDA) were mRNA vaccines from BioNTech/Pfizer and Moderna, demonstrating over 90% protective efficacy against symptomatic SARS-CoV-2 infection in phase III clinical trials [6, 7].

The success of mRNA vaccines was credited not just to their rapid design and development but also to their potent immune responses and overall safety [8]. mRNA constructs can be designed within a matter of days, facilitating fast production and scalability [9, 10]. Additionally, mRNA vaccines offer a safer alternative to live viruses and do not require access to the nucleus to depict their function, unlike deoxyribonucleic acid (DNA) vaccines [11]. Nanotechnology has been the solution to protect the RNA molecules and enable their intracellular delivery [12]. However, a challenge remains, which is the one associated to the necessity of intramuscular injection. The modality of intranasal vaccination would be an excellent alternative, as this method not only stimulates systemic immunity but also provides protection at the site of infection via the induction of mucosal immunity [13]. Additionally, this needle-free administration would be particularly relevant from a global perspective [14, 15]. Our lab has been among the pioneers on nanoparticles-based nasal vaccination and has developed a number of preclinical protein/peptide candidates for nasal protein administration [16–18]. In the area of RNA vaccination using synthetic drug delivery carriers, the advances so far have been relatively minorFor example, lipid-coated poly(β-amino ester) (PBAE)-based nanoparticles have been shown to successfully deliver luciferase mRNA (mLuc) to the nasal epithelium, resulting in significantly higher bioluminescence signal over naked mLuc [19]. In another instance, a cationic cyclodextrin-polyethyleneimine 2k conjugate complex was used to intranasally administer mRNA encoding for the human immunodeficiency virus (HIV) glycoprotein 120, leading to enhanced mucosal and systemic immune responses, as well as significant cytokine production [20]. Finally, only preliminary work has been reported on the use of LNPs for mRNA vaccination via intranasal administration against SARS-CoV-2, leading to certain levels of immunogenicity and protection against viral infection [21].

Since the beginning of the COVID pandemic, our laboratory has been part of a large consortium project oriented towards developing a SARS-CoV-2 vaccine. Our specific contribution to this project aimed at leveraging our expertise in nanocarriers for nucleic acid delivery and nanovaccines and developed a library of potential candidates for mRNA vaccination. Our ultimate goal was not only to protect the mRNA construct and to enhance overall immunogenicity but also to develop a formulation suitable for nasal administration. In our attempt to find an alternative to lipid nanoparticles (LNPs), we opted for a delivery platform extensively investigated in our lab for drug delivery consisting of nanoemulsions (NEs) and nanocapsules (NCs) and adapted it to the delivery of mRNA molecules vaccination [16–18, 22–25]. Based on our experience, NEs and NCs could be used as an alternative to LNPs, due to their composition based on regulatory acceptable biomaterials and simplicity, allowing them to be easily translated to a global complex.

Within this framework, we investigated various lipids, surfactants, oils, and polymers and produced a library of formulations with different compositions and physicochemical properties, all of them compiling with a target product profile (TPP), namely (A) particle size below or around 200 nm; (B) uniform particle size distribution; (C) high mRNA association capacity; (D) stability or lyophilization potential; and (E) alignment with regulatory requirements. Candidates meeting these criteria underwent in vitro and in vivo assessments of their transfection efficiency using model mRNAs. In vitro cytotoxicity and transfection efficiency were evaluated in HeLa cells, while initial in vivo studies included transfection efficiency evaluation upon intramuscular administration. These early studies led to the identification of a polymeric NC (NC-4-DX) and the optimization of a new generation NE (NE-13), containing both a cationic and an ionizable lipid. These candidates were them investigated for their immune responses following intranasal administration.

Overall, this article shows the formulation efforts to develop and optimize alternatives to LNPs for the purpose of mRNA vaccination in the context of the COVARNA consortium, including immunologists, pharmacologists, computer scientists, and molecular experts. Beyond the work presented here, focused on the development aspect of the formulations, within this consortium, different mRNAs intended to induce responses against the receptor binding domain (RBD) of the surface spike glycoprotein of the SARS-CoV-2 were developed [26]. These results have recently been published, highlighting the considerable differences in the SARS-CoV-2-specific neutralizing antibodies production depending on the nanoformulation [27].

## Materials and methods

### Materials

DOTAP (1,2-dioleoyl-3-trimethylammonium-propane, chloride salt) and DOPE (1,2-dioleoyl-sn-glycerol-3-phosphoethanolamine) were purchased from Avanti Polar Lipids (AL, USA). Vitamin E (Vit E) (D, L-α-tocopherol) was obtained from BASF (Mannheim, Germany). Tween^®^ 80, sucrose, and glucose (D(+)-glucose) were purchased from Merk KGaA (Darmstadt, Germany). Captex8000NF (caprylic acid triglycerides) was obtained from ABITEC Corporation (OH, USA). Protamine (PR) (protamine sulfate ED) was purchased from Yuki Gosei Kogyo (Tokio, Japan). Dextran sulfate (DX), sodium salt, was purchased from Sigma-Aldrich SAFC^®^ (MO, USA). Chitosan (CS) (poly (D-glucosamide) hydrochloride salt) was obtained from HMC^+^ (Halle, Germany). PEG (5 kDa)-b-PGA (10) [Na] (PEG-PGA or PP) (poly(ethylene glycol)-block-poly(l-glutamic sodium salt) was obtained from Polypeptide Therapeutic Solution (Valencia, Spain). C12-200 (1,1′-((2-(4-(2-((2-(bis(2-hydroxydodecyl)amino)ethyl)(2-hydroxydodecyl)amino)ethyl)piperazin-1-yl)ethyl)azanediyl)bis(dodecan-2-ol)) was purchased from Corden Pharma GmbH (Plankstadt, Germany).

mRNA encoding for green fluorescent protein (mGFP), luciferase (mLuc), and ovalbumin (mOVA) were kindly provided by Prof. Thieleman’s Lab, from Vrije University of Brussels (Brussels, Belgium). RPMI-1640 cell culture medium, phorbol 12-myristate 13-acetate (PMA), and ionomycin were purchased from Merck (Darmstadt, Germany). Ficoll Paque Plus, RBC lysis buffer, fetal bovine serum (FBS), penicillin/streptomycin (10.000 U/ml), L-glutamine 200 Mm, were obtained from ThermoFisher (CA, USA). Anti-mouse CD45-BV605 and BV605 Rat IgG2b, *k* isotype control, were purchased from BD (NJ, USA). Anti-mouse CD8-FITC and FITC Rat IgG2a, *k* isotype control, were obtained from BioLegend (CA, USA). OVA peptide was purchased from GenScript (NJ, USA). MHC I Dextramer OVA-specific antigen (JD2164-PE) was obtained from Immudex, (Virum, Denmark). Murine IFN-γ ELISpot Set was purchased from Diaclone (Besançon, France).

### Formulation of nanoemulsions

Two different approaches were used for the preparation of nanoemulsions (NEs): bulk mixing of cationic, blank NEs followed by the electrostatic complexation of mRNA onto the surface; or microfluidic production of NEs in the presence of mRNA, in a single step, using a microfluidic mixer system.

The preparation method for blank NEs consisted of a solvent-displacement technique [28]. An organic phase (0.3 mL for NE-1 to NE-5 and 0.4 mL for NE-6 to NE-14) was prepared by dissolving the appropriate amount of different lipids (including DOTAP, phospholipids, oils, or surfactants) in ethanol. The resulting solutions were added over an aqueous phase (2 mL, RNase-free water), under magnetic stirring (1400 rpm), and further incubated under stirring for 5 min. In the particular case of NE-15, blank NEs were prepared by microfluidic mixing, following the same protocol as described below in the absence of mRNA in the aqueous phase. The resulting blank NE was allowed to stabilize for at least 10 min. Complexation of mRNA onto the pre-formed blank NEs was performed by bulk mixing. Shortly, blank NE was diluted in RNase-free water, reaching the desired DOTAP concentration depending on the nitrogen-to-phosphate (N/P) ratio explored and the desired final mRNA concentration. A solution containing mRNA (at different concentrations, depending on the desired mRNA concentration and N/P ratio) was added over the previously formed blank NE solution, under magnetic stirring at 700 rpm for 10 s. The resulting NE-mRNA formulations were allowed to stabilize for 30 min. Different N/P ratios were explored, from 0.64:1 to 4:1. Volume-to-volume (v/v) between the mRNA and the blank NE was 2:1 for NE-1 to NE-5 and NE-13, and 4:1 for NE-6 to NE-12.

The formulation method based on microfluidic mixing comprises the simultaneous formation of the NE and the complexation of the mRNA onto a micromixer NanoAssemblr™ bench-top instrument, Precision NanoSystems Inc. (Vancouver, Canada). In summary, an organic phase (consisting of DOTAP, phospholipids, oils, or surfactants) and an aqueous phase (containing mRNA at the desired concentration) were simultaneously mixed into the micromixer. The aqueous-to-ethanol flow rate was set as 5:1, and the total flow rate was 12 mL/min. The different N/P ratios were maintained as described in the bulk mixing method. The resulting NE-mRNA formulations were allowed to stabilize for 30 min.

### Formulation of nanocapsules

NCs were produced by the coating of NE-mRNA with different polymers, including protamine (PR), chitosan (CS), dextran sulfate (DX), and PEG-PGA. Briefly, a polymeric solution (at a concentration-dependent on the weight-to-weight (w/w) ratio and the type of polymer used) was added over the previously formed NE-mRNA solution, under magnetic stirring at 700 rpm for 20 s. The resulting NC-mRNA carriers were stabilized for 30 min. The v/v ratio between the polymer and the NE-mRNA was 5:1.

### Physicochemical characterization of nanoemulsions and nanocapsules

Hydrodynamic diameter and polydisperse index (PDI) of the NEs and NCs were characterized by dynamic light scattering (Zetasizer^®^ Nano ZS, Malvern Instruments, Malvern, UK). ζ-potential was measured in terms of mean electrophoretic mobility values, measured by laser Doppler electrophoresis with the same equipment. Particle size and PDI measurements were performed after diluting the samples 10x in RNase-free water. ζ-potential characterization was performed after dilution of samples 20x in RNase-free water.

Encapsulation efficiency (EE%) was determined by a gel mobility assay using agarose gel electrophoresis. Briefly, both NE-mRNA and NC-mRNA formulations were diluted in a 1:1 (v/v) ratio with a solution of heparin (Sigma-Aldrich, MO, USA) or Triton X (Triton-X-100, Sigma-Aldrich, MO, USA), prepared at 50 mg/mL in RNase-free water, intended to displace the mRNA from the nanoparticles. Then, samples containing 1–3 µg of mRNA were loaded in an agarose gel at 1% w/v in Tris Acetate-EDTA buffer (Sigma-Aldrich, MO, USA) before and after incubation with heparin or Triton X solution. Samples were diluted with equal volumes of loading mix, containing 1x SYBR^®^ Gold nucleic acid strain (Invitrogen, CA, USA). Free mRNA was included as a control. Gels were run for 30 min at 90 V in a Sub-Cell GT cell 96/192 (Bio-Rad Laboratories, CA, USA), and evaluated with a UV transilluminator imaging system (Molecular Imager^®^ Gel Doc™ XR, Bio-Rad Laboratories, CA, USA).

### Freeze-drying of selected nanoemulsions and nanocapsules

Selected NE-mRNA and NC-mRNA formulations were frozen in the presence of cryoprotectants (including sucrose, glucose, and trehalose) at -40 ºC for at least 1 h, and subsequently freeze-dried (Genesis™ 25 EL, S.P Industries, PA, USA). Samples were initially left freeze-dried at -65 ºC for 1 h with a vacuum of 200 mTorr, to ensure that formulations were completely frozen. The first drying phase was performed from − 40 ºC to 15 ºC, under a progressive vacuum to 20 mTorr for 20 h. The second drying phase was done for 1 h at 20 ºC and 20 mTorr. After this process, formulations were stored at 4 ºC until resuspension in RNase-free water, and their physiochemical properties were determined.

### In vitro assessment of transfection efficiency and cytotoxicity of nanoemulsions and nanocapsules containing mRNA GFP

A total of 10,000 HeLa cells were seeded per well in a flat bottom 96-well plate and allowed to adhere for 24 h. Cells were treated with NE-mGFP and NC-mGFP formulations for 4 h, in Opti-MEM™ (Gibco™, Themo Fisher, MA, USA) at mGFP concentration ranging from 200 to 25 ng per well. Formulations were then removed, and replaced with complete medium, and cells were incubated for another 20 h. Cell viability was measured by resazuring assay, following manufacturer recommendations [29]. In brief, cells were incubated with resazuring reagent (Resazurin sodium salt, Sigma-Aldrich, MO, USA) supplemented with complete media for 45 min. Fluorescence was measured in a plate reader at excitation/emission wavelength of 544/590 nm. Cells were trypsinized, harvested, and fixed with 1% (w/v) formaldehyde in PBS, for flow cytometry analysis in terms of the percentage of GFP-positive cells and mean fluorescence intensity.

In other experiments, 60,000 HeLa cells per well were seeded in 24-well plate. In these experiments, mGFP concentrations of up to 1000 ng were used.

### Animal studies

All animal procedures were performed under the approved animal protocols 15,012/14/001 and 15,012/2023/004 (Consellería de Medio Rural, Xunta de Galicia), following National (RD 53/2013) and European (Directive 2010/63/EU) normative. Efforts were made to minimize the suffering of the animals used.

### In vivo transfection efficiency with mRNA Luc encapsulated onto nanoemulsions and nanocapsules following intramuscular administration

Swiss mice were administered two intramuscular injections (one in each thigh muscle), in a single administration session, 10 µg of mLuc, encapsulated in different NE-mLuc and NC-mLuc. The total volume administered was 50 µg per leg. This led to a total volume of 100 µL with a total dose of 20 µg per animal. At different time points, including 6, 24, and 48 h, animals were intraperitoneally administered 100 µL of D-luciferin, and whole-body fluorescence was visualized in an in vivo imaging system (IVIS).

### In vivo inoculation of mRNA OVA encapsulated onto nanoemulsions and nanocapsules following intranasal administration

#### Intranasal administration and sampling

C57BL/6J female mice (6–8 w/o) were purchased from Centro de Biomedicina Experimental CEBEGA (Universidade de Santiago de Compostela). Animals were fed *ad libitum* and housed in DVC (digital ventilated cages) with 12/12 h dark/light cycle. Intranasal administration was performed by specialized personnel in 3 mice groups: (1) free OVA mRNA (control, *n* = 6–9); (2) NE-13 formulation (*n* = 7–8) and (3) NC-4-DX formulation (*n* = 8). A total volume of 50 µL (50 µg mRNA) was administered intranasally at days 0 and 7, following the administration protocol depicted in Supplementary Fig. 1. To perform Dextramer staining and flow cytometry, peripheral blood samples (150 µL approx.) were obtained in lithium heparin tubes (Sarstedt™) from the submandibular vein on day 7 of the experiment. Blood samples were diluted in sterile PBS (ratio 1:2, blood: PBS) and PBMCs monolayer was obtained after Ficoll (ratio 4:5, Ficoll: diluted blood) centrifugation (400G, 25 min, room temperature). Mice were sacrificed at day 10 with CO_2_ and spleens were kept in sterile PBS. Splenocytes were obtained by mechanical digestion by filtering them through a 40 μm nylon filter. After two washes with PBS, cells were centrifuged (300G, 5 min, room temperature) and the supernatant was resuspended with 2 ml RBC lysis buffer for 2 min. Cells were then resuspended in 15 mL of PBS, centrifuged (300G, 5 min, room temperature), and resuspended in 10 mL RPMI.

#### Flow cytometry

To identify CD45^+^/CD8^+^/Dextramer^+^ populations, cells were stained with MHC Dextramer-PE for 10 min at room temperature. Afterward, antiCD45-BV605 and antiCD8-FITC were incubated for 20 min at room temperature. Corresponding rat IgG2b-BV605 and rat IgG2a-FITC isotypes were used as controls. Cell acquisition was performed in Accuri’s flow cytometer (BD) whereas data analysis was carried out in FlowJo™ platform.

#### Murine IFNƴ ELISpot

Splenocytes (2 × 10^5^ cells/condition) were plated in 96-well plates with RPMI + 10% FBS + 1% Penicillin/Streptomycin. Cells were stimulated overnight with 10 µg/mL OVA peptide and ELISpot kit was performed following the manufacturer’s instructions. Corresponding negative (unstimulated) and positive (1 ng/mL PMA + 0.5 µg/mL ionomycin) controls were used. The plate was read with Mabtech© instrument and analyzed with Mabtech© Apex Version 2.0.56.186.

## Results and discussion

### Screening of nanoemulsions (NEs) and nanocapsules (NCs)

As indicated, in this work we leverage the decades of experience of our lab in the use of polymers and lipids for the delivery of nucleic acids [16–18, 22–25]. Hence, we developed a library of NEs and polymeric NCs for the efficient delivery of mRNA, as a first step to the development of nasal formulations. For the selection of adequate candidates, we proposed a TPP a Target Product Profile with several requirements, including (A) particle size below 200 nm, preferably close to 100 nm; (B) uniform particle size distribution; (C) high mRNA association capacity; (D) stability during storage or lyophilization potential; and (E) alignment with regulatory requirements (e.g. low ethanol content, or the use of compounds already approved).

In the case of NEs, different combinations and molar ratios between the different components were explored, as depicted in Table 1. These nanocarriers were composed of a combination of cationic or ionizable lipids (DOTAP and C12-200, respectively), a helper lipid (DOPE), an oil (Vitamin E or Captex800 NF), and a surfactant (Tween^®^ 80). These different lipidic components were combined in different proportions and the resulting prototypes were analyzed in terms of mRNA encapsulation, particle size, colloidal stability, or transfection efficiency.

**Table 1:** Summary of the lipid compositions and molar ratios between the different components of the nanoemulsions investigated

Code	Lipid composition	Molar composition (%)
NE-1	DOTAP: DOPE: Vit E	16.8: 15.7: 67.5
NE-2		40.7: 11.2: 48.1
NE-3	DOTAP: DOPE: Vit E: T80	38.1: 10.5: 45.1: 6.4
NE-4		17.3: 8.1: 69.7: 4.9
NE-5		46.2: 9: 35.6: 9.3
NE-6	DOTAP: Captex8000 NF: T80	29: 43: 28
NE-7	DOTAP: DOPE: Captex8000 NF	22.5: 10.6: 66.9
NE-8	DOTAP: DOPE: Captex8000 NF: T80	25.5: 12: 37.8: 24.7
NE-9		21.1: 9.9: 59.7: 9.4
NE-10		29.9: 8.8: 53: 8.3
NE-11	DOTAP: C12-200: DOPE: Captex8000 NF: T80	20.4: 9.6: 3.1: 57.8: 9.1
NE-12		19.8: 6.1: 9.3: 56: 8.8
NE-13		4: 16: 10: 60: 10

**Abbreviations**: C12-200: 1,1’-((2-(4-(2-((2-(bis(2-hydroxydodecyl)amino)ethyl) (2-hydroxydodecyl)amino) ethyl)piperazin-1-yl)azanediyl)bis(dodecan-2-ol) DOPE: 1,2-dioleoyl-sn-glycero-3-phosphoethanolamine. DOTAP: 1,2-dioleoyl-3-trimethylammonium propane. NE: nanoemulsion. T80: Tween^®^ 80. Vit E: D, L-α-tocopherol

Following mRNA entrapment, these NE-mRNA carriers were used as a template for the incorporation of a polymer shell driven by electrostatic assembling. This polymer shell was expected to modify the interaction of the nanocarrier with the nasal epithelium. Based on our previous work of chitosan (CS), protamine (PR), and dextran sulphate (DX) vaccine delivery carriers [17, 18, 22, 24, 25], we selected these polymers as well as the PEG-PGA (PP), extensively used in our laboratory for other applications. Besides our own work, these polymers have previously been used as adjuvant and antigen delivery systems, capable of activating different T and B cell responses [30–34]. As detailed in Table 2, the nomenclature for the resulting NC-mRNAs was derived from the initial NE name code, for easy identification of the core composition.

**Table 2:** Summary of the lipid and polymeric compositions, and the molar and weight-to-weight ratio between the different components of the nanocapsules investigated

Code	Initial NE	Molar composition (%)	Polymer coating	mRNA: polymer (w/w)
NC-1-PR	DOTAP: DOPE: Vit E	16.8: 15.7: 67.5	Protamine	1:1
NC-3-PR	DOTAP: DOPE: Vit E: T80	38.1: 10.5: 45.1: 6.4		1:1
NC-4-PR		17.3: 8.1: 69.7: 4.9		1:1
NC-7-PR	DOTAP: DOPE: Captex8000 NF	22.5: 10.6: 66.9		1:1
NC-8-PR	DOTAP: DOPE: Captex8000 NF: T80	25.5: 12: 37.8: 24.7		1:1
NC-9-PR		21.1: 9.9: 59.7: 9.4		1:1
NC-3-DX	DOTAP: DOPE: Vit E: T80	38.1: 10.5: 45.1: 6.4	Dextran Sulphate	1:1
NC-4-DX		17.3: 8.1: 69.7: 4.9		1:1 or 1:2
NC-5-DX		46.2: 9: 35.6: 9.3		1:2
NC-3-CS	DOTAP: DOPE: Vit E: T80	38.1: 10.5: 45.1: 6.4	Chitosan	1:1
NC-4-CS		17.3: 8.1: 69.7: 4.9		1:1
NC-4-PP	DOTAP: DOPE: Vit E: T80	17.3: 8.1: 69.7: 4.9	PEG-PGA	1:12
NC-5-PP		46.2: 9: 35.6: 9.3		1:12

**Abbreviations**: CS: chitosan. DOPE: 1,2-dioleoyl-sn-glycero-3-phosphoethanolamine. DOTAP: 1,2-dioleoyl-3-trimethylammonium propane. DX: dextran sulphate. NC: nanocapsule. NE: nanoemulsion. PEG-PGA or PP: PEG (5 kDa)-b-PGA (10) Na. PR: protamine. T80: Tween^®^ 80. Vit E: D, L-α-tocopherol. w/w ratio: weight-to-weight ratio between polymer and mRNA content

#### Preparation of mRNA-loaded nanoemulsions (NEs)

NE-mRNAs were prepared using two distinct strategies. In the first approach, blank NEs were prepared by solvent-displacement technique, resulting in nanodroplets with a size in the range of 100 to 130 nm and a highly positive surface charge (Table 3). In a second step, mRNA was complexed onto the NE by electrostatic interactions between the positive charge of the cationic lipid and the negative charge of the nucleic acid, causing a rise in the particle size of the resultant NE-mRNAs. Furthermore, a reduction in the PDI was noted following mRNA absorption, attributed to the enhanced stabilization of the nanosystem through electrostatic complexation of the mRNA on the surface of the NEs. This contributed to the overall improvement in the stability and dispersity of the resulting nanocarrier. On the other hand, certain prototypes were prepared in a single-step microfluidic mixing of both aqueous and organic phases, leading to the simultaneous formation of the NEs and the complexation of mRNA. These two formulation strategies were performed with different mRNAs, including mGFP, mLuc, and mOVA, leading to different physicochemical characteristics (Table 4). Overall, the use of microfluidic mixing led to the formation of nanostructures with a smaller particle size (60–100 nm) than the bulk mixing of components (100–150 nm). These variations might suggest potential changes in the internal structure of the NEs and the organization of the mRNA molecules, potentially due to the rapid mixing enabled by the microfluidic device. In fact, this has been shown for certain compositions of LNPs [35–37].

**Table 3:** Physicochemical properties of blank NEs

Code	Size (nm)	PDI	Z-Pot (mV)
NE-3	102 ± 13	0.40 ± 0.05	+ 56 ± 5
NE-4	104 ± 15	0.30 ± 0.04	+ 55 ± 3
NE-9	108 ± 9	0.15 ± 0.03	+ 56 ± 8
NE-11	113 ± 6	0.20 ± 0.01	+ 58 ± 2
NE-12	131 ± 15	0.16 ± 0.01	+ 56 ± 2

**Abbreviations**: NE: nanoemulsion. PDI: polydispersity index. Values represent the mean ± standard deviation (*n* ≥ 3, unless indicated otherwise)

**Table 4:** Physicochemical properties of NE-mRNA nanocarriers, using different types of mRNAs and different N/P ratios

Code	Type of mRNA	Formulation method	*N*/*P* ratio	Size (nm)	PDI	ζ-Potential (mV)	EE (%)
NE-3	mGFP	Bulk mixing	2:1	108 ± 13	0.14 ± 0.03	+ 39 ± 2	100
NE-4	mGFP	Bulk mixing	2:1	127 ± 13	0.09 ± 0.03	+ 43 ± 4	100
	mLuc	Bulk mixing	2:1	138 ± 18	0.14 ± 0.06	+ 42 ± 2	100
	mLuc	Bulk mixing	4:1	89 ± 2	0.21 ± 0.02	+ 48 ± 6	100
	mLuc	Microfluidics	4:1	84 ± 11	0.21 ± 0.02	+ 54 ± 3	100
	mOVA	Bulk mixing	2:1	121 ± 9	0.10 ± 0.01	+ 40 ± 3	100
NE-5	mGFP	Microfluidics	4:1	62 ± 13	0.24 ± 0.07	+ 53 ± 7	100
NE-9	mGFP	Bulk mixing	4:1	97 ± 1	0.19 ± 0.02	+ 51 ± 3	100
	mLuc	Bulk mixing	4:1	104 ± 1	0.17 ± 0.01	+ 52 ± 2	100
NE-10	mGFP	Microfluidics	4:1	65 ± 1	0.2 ± 0.01	+ 44 ± 1	100
NE-11	mGFP	Bulk mixing	4:1	107 ± 3	0.18 ± 0.02	+ 53 ± 2	100
NE-12	mGFP	Bulk mixing	4:1	123 ± 2	0.17 ± 0.01	+ 49 ± 1	100
NE-13	mOVA	Microfluidics	2:1	116 ± 1	0.17 ± 0.01	+ 49 ± 1	75

**Abbreviations**: EE: encapsulation efficiency. mGFP: mRNA encoding for GFP. mLuc: mRNA encoding for luciferase. mOVA: mRNA encoding ovalbumin protein. NE: nanoemulsion. N/P ratio: nitrogen to phosphate ratio. PDI: polydispersity index. Values represent the mean ± standard deviation (*n* ≥ 3, unless indicated otherwise)

Furthermore, as expected, the incorporation of negatively charged mRNA onto the surface of the previously formed blank NEs led to a significant reduction of their positive charge. The resulting surface charges of NE-mRNAs were slightly negative at low N/P ratios, and highly positive at high N/P ratios. The N/P ratio also influenced the EE% values, with higher values obtained at a higher N/P ratio. For example, NE-1, -2, -6, -7, and − 8 were prepared at an N/P ratio of 0.6:1, leading to particle sizes between 115 and 150 nm, negative surface charges (-5 to -10 mV), and low EE% values (below 50% in all cases). Additionally, different types of mRNA exhibited different particle diameters and PDI due to their distinct interaction with the cationic DOTAP lipid.

Selected NE-mRNAs were subjected to a lyophilization process, intended to preserve the physicochemical properties of the nanosystems and the integrity of the mRNA cargo upon long-time storage (Supplementary Table 1). Different cryoprotectants, added at different percentages, were tested. In general, the incorporation of sucrose (10–20% w/v) allowed the maintenance of the physicochemical properties of the NE-mRNAs upon resuspension, suggesting the possibility of long-term storage for these nanocarriers.

#### Preparation of mRNA-loaded nanocapsules (NCs)

The resulting NE-mRNA nanocarriers were subsequently coated with various polymers to form a polymer shell, intended to modulate the surface properties of the resulting NC-mRNAs (Table 5). Different w/w ratios between the polymer and the mRNA were evaluated for each NC-mRNA nanosystem, based on the amount of polymer required to effectively modify the surface properties while preserving particle stability. The formation of the polymer shell around the oily nanodroplets led to significant changes in the physicochemical properties. In order to facilitate the interaction of polymers with NE-mRNAs formulations, the net charge of NE-mRNAs was adjusted by the N/P ratio. Namely, NC-PR-mRNAs and NC-CS-mRNAs were prepared at N/P ratios between 0.6:1 to 0.9:1, resulting in nanoparticles of 130–190 nm, low PDI and positive surface charges (+ 25 to + 30 mV), ensuring anionic surface charge on the NE-mRNAs and allowing the adequate absorption of the positively charged polymers.

**Table 5:** Physicochemical properties of NC-mRNA nanocarriers, using different types of mRNA, polymers and RNA to polymer ratios

Code	Type of mRNA	RNA: polymer ratio (w/w)	Size (nm)	PDI	Z-Pot (mV)	EE (%)
NC-3-PR	mGFP	1:1	152 ± 9	0.12 ± 0.01	+ 24 ± 1	100
NC-4-PR	mGFP	1:1	153 ± 16	0.11 ± 0.03	+ 27 ± 2	75
NC-3-DX	mGFP	1:1	144	0.09	-26	100
NC-4-DX	mLuc	1:1	154 ± 8	0.14 ± 0.04	-14 ± 1	100
		1:2	126 ± 3	0.11 ± 0.02	-21 ± 2	100
	mOVA	1:2	132 ± 3	0.07 ± 0.03	-16 ± 2	100
NC-5-DX_m_	mGFP	1:2	93 ± 9	0.2 ± 0.01	-6 ± 7	100
NC-5-PP_m_	mGFP	1:12	70 ± 1	0.17 ± 0.01	+ 13 ± 5	-

**Abbreviations**: CS: chitosan. DX: dextran sulphate. EE: encapsulation efficiency. _m_: base NE-mRNA prepared by microfluidics. mGFP: mRNA encoding for GFP. mLuc: mRNA encoding for luciferase. mOVA: mRNA encoding ovalbumin protein. NC: nanocapsule. PDI: polydispersity index. PP: PEG-PGA or PEG (5 kDa)-b-PGA (10) (Na). PR: protamine sulphate EP. w/w ratio: weight-to-weight ratio between polymer and mRNA coating. Values represent the mean ± standard deviation (*n* ≥ 3, unless indicated otherwise)

Overall, the particle diameters observed in the NC-mRNA nanosystems ranged from 70 to 240 nm, primarily dependent on the method used for the initial preparation of NE-mRNAs. Significant differences were observed for different mRNA molecules (e.g. overall, particle size observed when mLuc is encapsulated is greater than when other mRNAs are used). This suggests a different interaction between the NE, the mRNA and the polymer, mainly dependent on the type of mRNA used.

Selected NC-mRNAs were lyophilized as an assessment of their long-term preservation upon storage in these conditions (Supplementary Table 1). After screening of different cryoprotectants, a preliminary preference by certain polymers was observed. For example, NC-PR-mRNAs were capable of maintaining their physicochemical properties when using trehalose 10%, while this behavior was observed in NC-DX-mRNAs when lyophilized with sucrose 10%.

### In vitro assessment of cytotoxicity and transfection efficiency of NE-mGFP and NC-mGFP

HeLa cells were selected as a model for easy transfection and evaluation of cellular toxicity. These cells have also been used in different preclinical vaccine studies [38, 39]. Different NE-mGFP and NC-mGFP candidates were incubated with HeLa cells for 4 h, and toxicity and efficient translation into the florescence reporter protein were assessed 24 h after transfection.

**Fig. 1:**
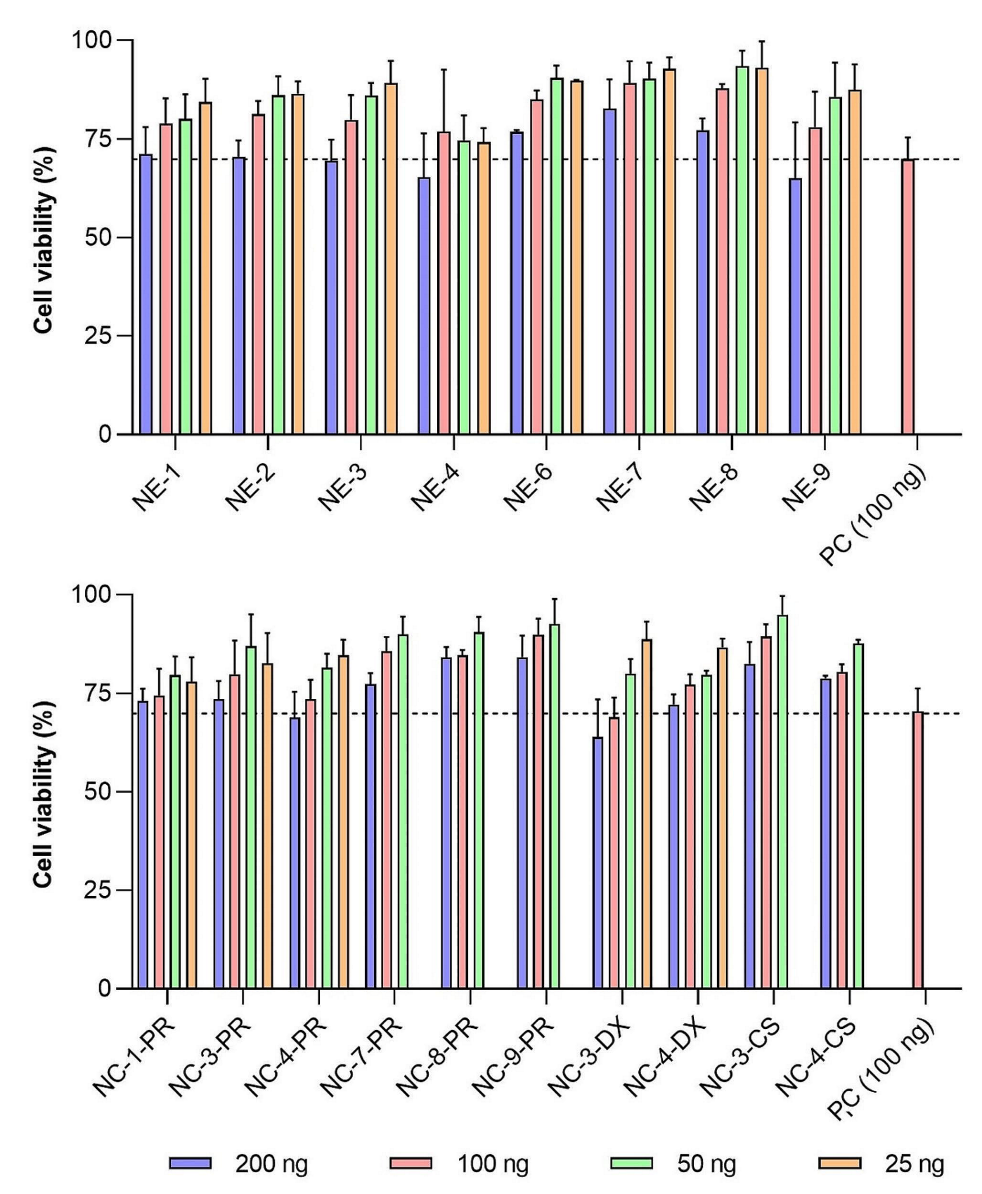
Citotoxicity of NE-mGFP (top) and NC-mGFP (bottom), at different mGFP concentrations, in HeLa cells at 24-hours post transfection Abbreviations: CS: chitosan. DX: dextran sulphate. mGFP: mRNA encoding for GFP. NE: nanoemulsion. NC: nanocapsule. PC: positive control, lipofectamine. PR: protamine sulphate EP. Values represent the mean ± standard deviation (*n* ≥ 3)

The results depicted in Fig. 1 show concentration-dependent toxicity in the range evaluated, with a maximum reduction in viability of approximately 25% observed at the highest doses investigated (200 and 100 ng of mRNA) for most of the tested nanosystems. No significant differences in cell viability were observed among the different NE-mGPF and NC-mGFP formulations tested. Notably, NC-mGFP exhibited slightly lower toxicity compared to NE-mGFP (for instance, NC-4-PR, NC-4-DX, and NC-4-CS resulted in better cell viability profiles than NE-4; and NE-9 showed lower viability than NC-9-PR). These findings suggest that the polymeric shell surrounding the NE-mGFP to form the NC-mRNA prototypes contributes to reducing the overall high surface charge of the NE-mGFP. Additionally, NC-mGFP with more neutral surface charges has the potential to yield better cell viability profiles. This reduction of cellular viability, primarily driven by the positive charge, aligns with previously reported findings [40, 41].

**Fig. 2:**
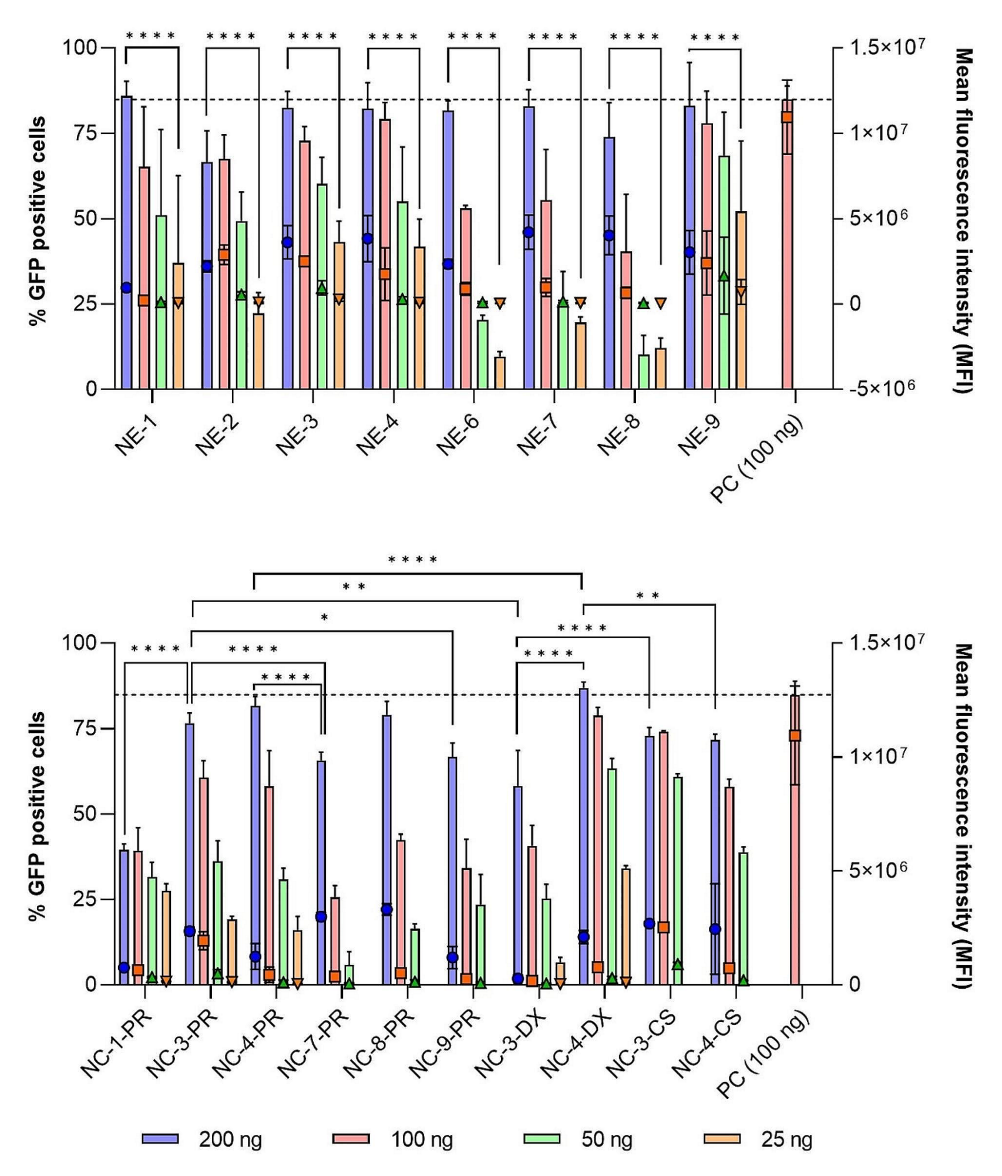
GFP transfection efficiency of NE-mGFP (top) and NC-mGFP (bottom) nanocarriers, at different mGFP concentrations. Transfection efficiency is expressed in percentage of GFP positive cells (bars, left axis) and mean fluorescence intensity (symbols, right axis) in HeLa cells, 24 h post transfection **Abbreviations**: CS: chitosan. DX: dextran sulphate. mGFP: mRNA encoding for GFP. NE: nanoemulsion. NC: nanocapsule. PC: positive control, lipofectamine. PR: protamine sulphate EP. A significant comparison was performed using two-way ANOVA followed by Turkey’s multiple comparison tests between the highest concentration and lower concentration (top) and between the highest concentration of each group (bottom). *p*-values < 0.05 were considered statistically significant (*). Also, (**) if *p*-value < 0.01, and (****) if *p*-value < 0.0001. Values represent the mean ± standard deviation (*n* ≥ 3)

To evaluate transfection efficiency, two metrics were utilized: the percentage of GFP-positive cells, indicating the proportion of cells capable of expressing GFP (Fig. 2, bars); and mean fluorescence intensity (MFI), which quantifies the amount of fluorescence emitted by these cells (Fig. 2, symbols). In general, NE-mGFP formulations (depicted in Fig. 2, top) exhibited higher percentages of GFP-positive cells and MFI compared to NC-mGFP formulations, especially at the lower RNA concentrations (Fig. 2, bottom). In all instances, a notable dose-dependent increase in transfection efficiency was observed. Among the various NE-mGFP formulations, NE-3-mGFP, NE-4-mGFP, and NE-9-mGFP showed the highest transfection efficiency. This might be related to the presence of Tween^®^ 80 in their composition and, also, to the appropriate combination of DOTAP and DOPE [42, 43].

Interestingly, NC-mGFP formulations exhibited different behavior depending on the polymer shell, with different significances depending on the concentration tested. For instance, both NC-3-PR-mGFP and NC-3-CS-mGFP exhibited superior percentages of GFP-positive cells compared to NC-3-DX-mGFP at the highest concentration tested. Furthermore, significant differences were observed among NC-PR formulations. NC-3-PR-mGFP and NC-4-PR-mGFP resulted in greater GFP-positive cell levels than NC-1-PR-mGFP, NC-7-PR-mGFP, or NC-9-PR-mGFP. These findings indicate that the NE-mGFP used to form the NC-mGFP formulations have the potential to determine their final cellular transfection potential, possible due to the lipidic composition of the oily nanodroplets, driving the internal cellular fate of the nanocarriers. On the other hand, NC-4-DX-mGFP markedly outperformed NC-3-DX-mGFP, despite both utilizing DX as the polymeric shell. These disparities between nanosystems were not evident were comparing NE-3-mGFP and NE-4-mGFP, thus suggesting that the entanglement of the polymeric shell may influence the transfection efficiency of the nanocarriers.

Selected formulations, all prepared via microfluidic mixing, were tested in HeLa cells using a larger number of cells in 24-well plates, including NE-5-mGFP, NE-9-mGFP, NE-10-mGFP, NE-11-mGFP, NE-12-mGFP, NC-5-DX-mGFP, and NC-5-PP-mGFP (refer to Supplementary Fig. 2). No significant cellular toxicity was observed for none of the concentrations tested. Regarding transfection efficiency, notorious differences were found between NE-9-mGFP and NE-10-mGFP (containing DOTAP as complexing lipid) as compared with NE-11-mGFP and NE-12-mGFP (containing a combination of DOTAP and C12-200 as complexing agents). These results highlight that the combination of DOTAP with an ionizable lipid (such as C12-200) could have positive effects on the transfection efficiency of nanocarriers. C12-200 is a multi-tailed ionizable lipidoid known for its ability to adopt a cone-shaped structure, with the potential to enhance the disruption of endosomes upon cellular uptake [44, 45].

### In vivo evaluation of NE-mRNA and NC-mRNA as vaccine delivery systems following intramuscular and intranasal administration

Selected NEs-mRNAs and NC-mRNAs were evaluated in different in vivo studies intended to assess their potency as vaccine delivery systems (Table 6). Intramuscular administration was used to deliver mRNA encoding for luciferase (mLuc) to assess the transfection potency of our candidates in vivo. In the subsequent in vivo study, the immune response capacity of chosen nanocarriers was evaluated following intranasal administration, using mRNA encoding for ovalbumin (mOVA).

**Table 6:** Summary of the NE-mRNAs and NC-mRNAs used for in vivo evaluation

Code	Composition	Polymer	Intramuscular administration (mLuc)	Intranasal administration (mOVA)
NE-4	DOTAP: DOPE: Vit E: T80 (17.3: 8.1: 69.7: 4.9)		✓	
NE-9	DOTAP: DOPE: Captex8000 NF: T80 (21.1: 9.9: 59.7: 9.4)		✓	
NE-12	DOTAP: C12-200: DOPE: Captex8000 NF: T80 (19.8: 6.1: 9.3: 56: 8.8)		✓	
NE-13	DOTAP: C12-200: DOPE: Captex8000 NF: T80 (4: 16: 10: 60: 10)			✓
NC-4-DX	DOTAP: DOPE: Vit E: T80 (17.3: 8.1: 69.7: 4.9)	Dextran Sulphate	✓	✓
NC-4-PP	DOTAP: DOPE: Vit E: T80 (17.3: 8.1: 69.7: 4.9)	PGA-PEG	✓	
NC-5-PP	DOTAP: DOPE: Vit E: T80 (46.2: 9: 35.6: 9.3)	PGA-PEG	✓	

**Abbreviations**: C12-200: 1,1’-((2-(4-(2-((2-(bis(2-hydroxydodecyl)amino)ethyl) (2-hydroxydodecyl)amino) ethyl)piperazin-1-yl)azanediyl)bis(dodecan-2-ol) DOPE: 1,2-dioleoyl-sn-glycero-3-phosphoethanolamine. DOTAP: 1,2-dioleoyl-3-trimethylammonium propane. DX: dextran sulphate. mLuc: mRNA encoding for luciferase. mOVA: mRNA encoding ovalbumin protein. NE: nanoemulsion. NC: nanocapsule. PP: PEG-PGA or PEG (5 kDa)-b-PGA (10) (Na). T80: Tween^®^ 80. Vit E: D, L-α-tocopherol

#### Intramuscular administration of nanocarriers with mLuc

**Fig. 3:**
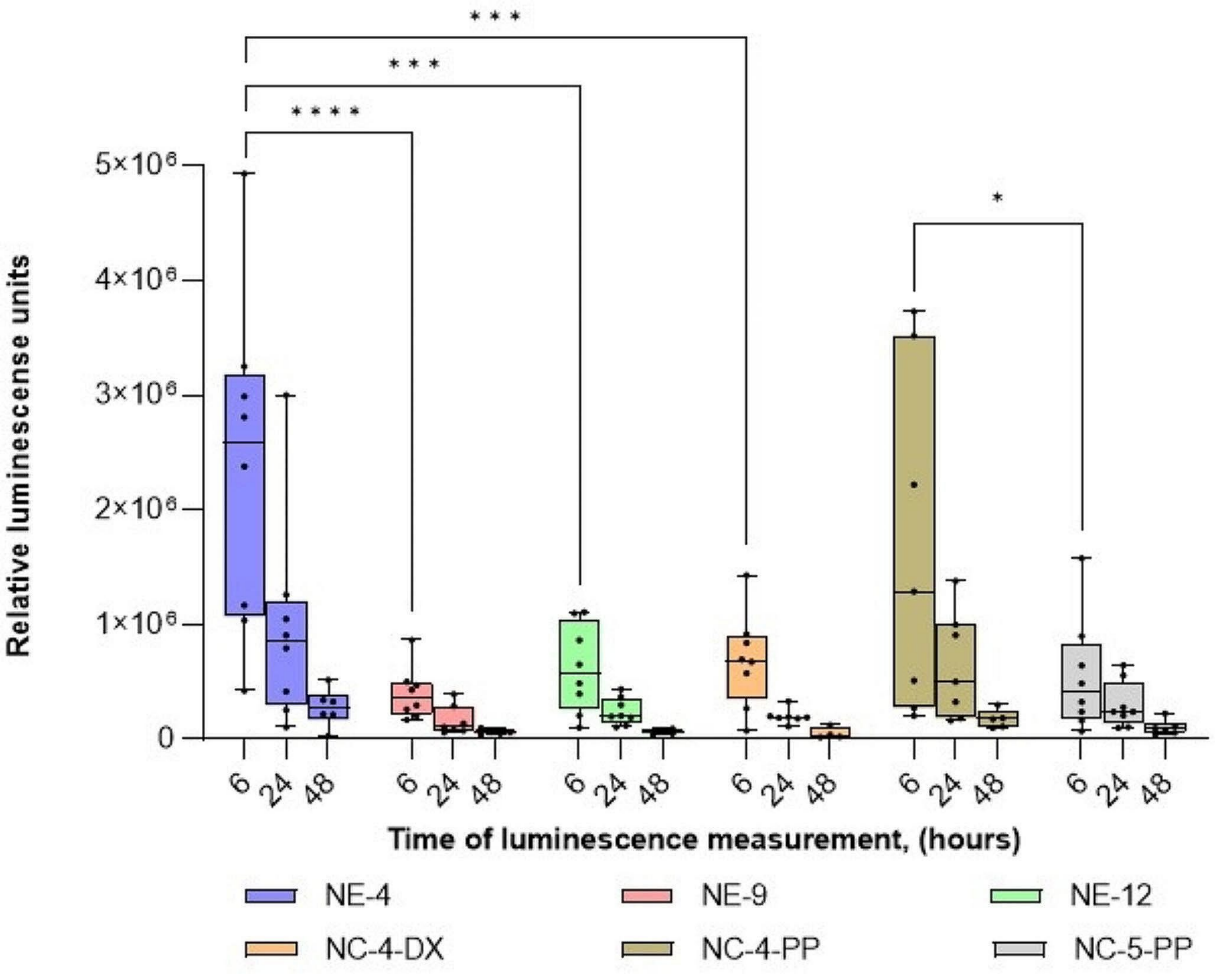
Quantification of whole-body luciferase signal of NE-mLuc and NC-mLuc formulations, after intramuscular administration. Each animal was injected in both legs and imaged by IVIS at different time points (6, 24, and 48 h) **Abbreviations**: DX: dextran sulphate. mLuc: mRNA encoding for luciferase. NE: nanoemulsion. NC: nanocapsule. PP: PGA-PEG or PEG (5 kDa)-b-PGA (10) (Na). A significant comparison was performed using two-way ANOVA followed by Turkey’s multiple comparison tests between the groups, at 6 h. *p*-values < 0.05 were considered statistically significant (*). Also, (***) if *p*-value < 0.001, and (****) if *p*-value < 0.0001. Values represent the mean ± standard deviation (*n* ≥ 3)

The preliminary assessment of the in vivo transfection efficiency of selected mRNA nanocarriers, was performed using mLuc. Figure 3, shows the expression of the reporter fluorescence protein following intramuscular administration in mice. Although a strict in vitro/in vivo correlation was not observed for all formulations tested (Fig. 2), the in vivo results showed that, among the NE-mLuc formulations tested (NE-4-mLuc, NE-9-mLuc, and NE-12-mLuc), NE-4-mLuc exhibited the highest capacity to produce luciferase In addition, when comparing formulations with the same polymer shell but different NE cores (e.g. NC-4-PP-mLuc and NC-5-PP-mLuc), some differences were observed. In line with the in vitro results (Fig. 2), even when using the most potent NE-mLuc (such as NE-4-mLuc), the choice of polymer can modulate the transfection efficiency of the resulting NC-mLuc nanocarrier to some extent, although not exceeding the performance of NE-mLuc.

The performance of our most potent nanocarriers (namely, NE-4-mLuc and NC-4-PP-mLuc prototypes) could be in relation to the appropriate combination of all ingredients (DOTAP, DOPE, Vitamin E, and Tween 80^®^, at molar ratio 17.3: 8.1: 69.7: 4.9). These performances could be improved by the use of more advanced complexing lipids, such as ionizable lipids, resulting in significant enhancement of the potency of mLuc transfection upon intramuscular administration [46, 47]. In summary, the potency of the nanocarriers can be fine-tuned by selecting high-performance lipids and more potent mRNA cargos.

IVIS images corresponding to this study can be found in Supplementary Fig. 3.

#### Immune response assessment of intranasal administration of NE-13 and NC-4-DX containing mOVA

Based on the synergy observed for the combination of DOTAP and C12-200 observed on in vitro studies, an optimized version of the previous NE-11 and NE-12 formulations was developed. In the earlier iterations, the quantity of DOTAP significantly outweighed that of C12-200. In the new optimized version, named NE-13, the proportion of C12-200 was substantially higher than that of DOTAP. This adjustment aimed to exploit the enhanced endosomal escape capabilities offered by ionizable lipids, such as C12-200. Upon cellular uptake, this lipid becomes protonated within acidic endosomes, thereby interacting with endosomal phospholipids. This interaction induces a phase transition from a highly stable bilayer structure to an inverted hexagonal H_II_ phase, capable of rupturing the endosomal membrane and facilitating the release of mRNA cargo into the cytosol [48].

On the other hand, based on the aforementioned in vitro and in vivo studies, NC-4-DX emerged as a promising nanocarrier without need of subsequent optimization, considering the potential immune responses elicited by dextran sulphate-based nanocarriers in the past [18, 24]. Its favorable cellular safety and transfection profile, both in vitro and in vivo, coupled with its slightly negative surface charge (in contrast to the highly positive surface charges observed in NE-mRNAs), position it as a promising candidate for further exploration.

These two selected candidates were utilized to encapsulate mOVA [49]. To evaluate in vivo immune responses induced by NE-13 and NC-4-DX, animals were intranasally administered twice (day 0 and 7).

**Fig. 4:**
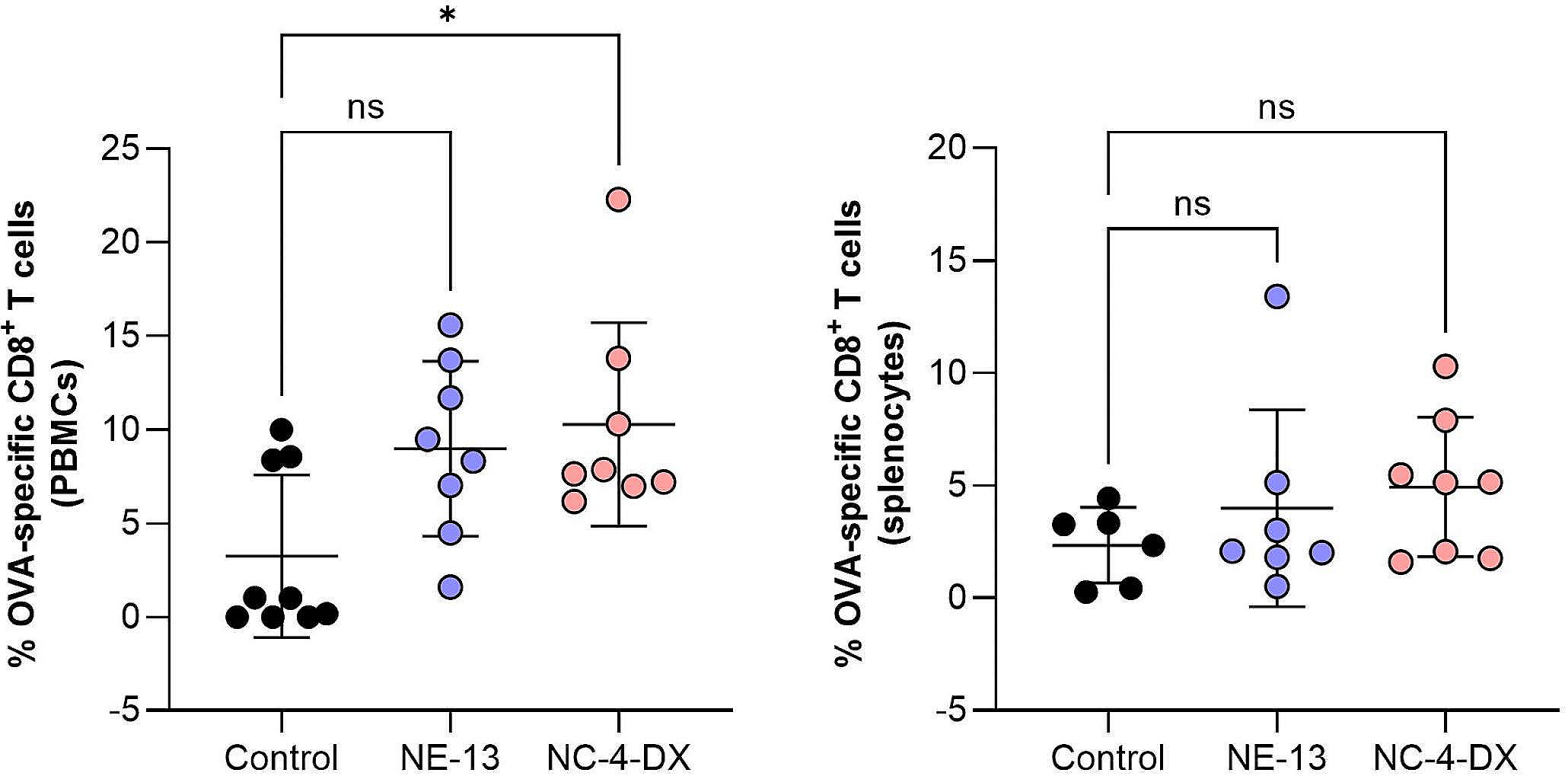
Percentage of OVA-specific CD8^+^ T cells upon intranasal administration con NE-13-mOVA and NC-4-DX-mOVA, obtained by flow cytometer, in blood (collected on day 7 post-administration, left) and splenocytes (collected on day 10 post-administration, right). The percentage of antigen-specific CD8^+^ T cells was gated as CD45^+^/CD8^+^/Dextramer^+^ **Abbreviations**: DX: dextran sulphate. mOVA: mRNA encoding for ovalbumin. NE: nanoemulsion. NC: nanocapsule. PBMCs: peripheral blood mononuclear cells. A significant comparison was performed using one-way ANOVA followed by Turkey’s multiple comparison tests between the groups. *p*-values < 0.05 were considered statistically significant. Values represent the mean ± standard deviation (*n* ≥ 3)

The adaptative immune system is designed to provide protection from recurring infections, and can be activated through humoral responses (mainly, antibodies) or cellular responses (T cells). Cellular response can induce specialized T cells, known as cytotoxic T cells (CTLs), or clusters of differentiation 8 (CD8^+^) T cells. Ideally, the nanosystems developed should be able to induce robust Major Histocompatibility Complex I (MHC-I)-mediated CD8^+^ T cell responses [50, 51]. To evaluate this desirable immune response, detection of antigen-specific CD8^+^ T cells was performed by MHC I Dextramer-PE staining in PBMCs (collected on day 7, Fig. 4, left) and splenocytes (collected on day 10, Fig. 4, right).

In blood samples, both formulations, NE-13-mOVA and NC-4-DX-mOVA, resulted in an increase in antigen-specific CD8^+^ T cell numbers (gated as CD45^+^/CD8^+^/Dextramer^+^), as compared with the control group (Fig. 4, left). However, although the percentage of antigen-specific CD8^+^ T cells is similar for both formulations, only NC-4-DX-mOVA has the capacity to induce a statistically significant increase. At day 10 post-administration, splenocytes were harvested and results showed a similar tendency as at day 7 (Fig. 4, right). Compared with the control group, both formulations induced an in vivo immune response by increasing the percentage of OVA-specific CD8^+^ T cells. Further, NC-4-DX-mOVA resulted in the formulation with the highest immunological effect, as previously observed for day 7.

In comparison to existing literature, there are only a limited number of examples of mRNA-lipid nanovaccines designed for intranasal delivery. A notable study in this realm is the preclinical investigation conducted by Moderna Inc., which employed mRNA-loaded LNPs for intranasal immunization against SARS-CoV-2. This study observed a substantial rise in antibody levels and a reduction in viral load within the respiratory tract. Nonetheless, cellular responses were not addressed in this research [21]. For anti-tumor proposes, the intranasal delivery of mRNA-loaded cationic liposomes demonstrated CD8^+^ T cell responses in splenocytes at approximately 5%, akin to the response observed with NC-4-DX-mOVA. In another instance focusing on anti-tumor immunity, mOVA nanoparticles were administered in both prophylactic and therapeutic rat models harboring OVA tumor cells [52]. In this study, the levels of OVA-specific CD8^+^ T cells in splenocytes were found to be 3%, which aligned closely with the levels seen with NE-13-mOVA and was lower compared to those achieved with NC-4-DX [53].

**Fig. 5:**
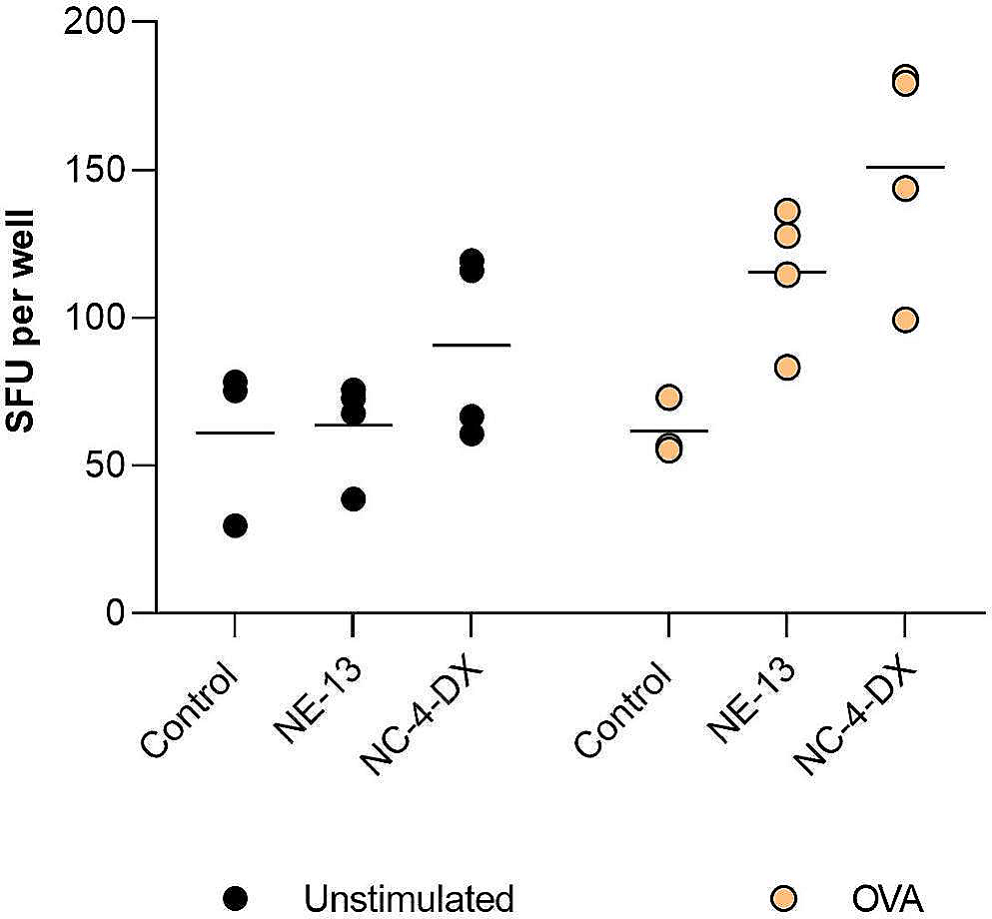
IFN-γ-producing spot-forming units (SFU) from splenocytes, by ELISpot assay (collected on day 10) after intranasal administration of NE-13-mOVA and NC-4-DX-mOVA. Study was performed in unstimulated (left) and OVA-stimulated splenocytes **Abbreviations**: DX: dextran sulphate. mOVA: mRNA encoding for ovalbumin. NE: nanoemulsion. NC: nanocapsule. A significant comparison was performed with mixed-effects analysis followed by Dunnett’s multiple comparison tests between the groups. All comparisons were not significant (*n* ≥ 3)

To measure CD8^+^ T cell responses, the quantity of interferon-gamma (IFN-γ) induced in splenocytes following intranasal administration of NE-13-mOVA and NC-4-DX-mOVA (Fig. 5). In accordance with blood sample results, immune cells were stimulated by both NE-13-mOVA and NC-4-DX-mOVA formulations, with the latter having a higher evident stimulatory effect. Further, IFN-γ production was higher after OVA stimulation than in unstimulated immune cells for both formulations.

Overall, these results indicated that both NE-13-mOVA and NC-4-DX-mOVA are promising delivery systems for mRNA vaccination, inducing robust CD8^+^ T cell activation. Furthermore, this study shows consistent cellular immune responses for both OVA-specific CD8^+^ T cell activation and IFN-γ release, determined by Dextramer and IFN- γ ELISpot assay, two powerful technologies for accurately assessing cellular immune responses after vaccination [54].

## Conclusions

In the present study, we engineered different NEs and polymeric NCs as mRNA vaccine candidates for intranasal administration. The results underscore the significant impact of multiple factors, including the components and molar ratios of the NEs, the identity of the polymeric shell of the NCs, and the combination of ionizable and cationic lipids, on the transfection efficiency and cytotoxicity profile of our nanocarriers. Notably, NC-4-DX resulted in the best-performing nanocarrier, capable of delivering the desired mRNA effectively via intramuscular administration and eliciting robust cellular immune responses following intranasal administration.

In summary, the composition, surface polymer modification, and the use of different types of lipids for mRNA entrapment may influence the performance of the mRNA vaccine.

### Electronic supplementary material

Below is the link to the electronic supplementary material.

Supplementary Material 1

Supplementary Material 2

Supplementary Material 3

Supplementary Material 4

## References

[1] Cucinotta D, Vanelli M (2020). WHO declares COVID-19 a pandemic. Acta Biomed.

[2] Hu B, Guo H, Zhou P, Shi ZL (2021). Characteristics of SARS-CoV-2 and COVID-19. Nat Rev Microbiol.

[3] Liu Y, Liu J, Plante KS, Plante JA, Xie X, Zhang X, Ku Z, An Z, Scharton D, Schindewolf C, Widen SG, Menachery VD, Shi PY, Weaver SC (2022). The N501Y spike substitution enhances SARS-CoV-2 infection and transmission. Nature.

[4] World Health Organization, WHO Coronavirus (COVID-19) Dashboard. (2023). https://covid19.who.int/ (accessed May 29, 2023).

[5] Corbett KS, Edwards DK, Leist SR, Abiona OM, Boyoglu-Barnum S, Gillespie RA, Himansu S, Schäfer A, Ziwawo CT, DiPiazza AT, Dinnon KH, Elbashir SM, Shaw CA, Woods A, Fritch EJ, Martinez DR, Bock KW, Minai M, Nagata BM, Hutchinson GB, Wu K, Henry C, Bahl K, Garcia-Dominguez D, Ma LZ, Renzi I, Kong WP, Schmidt SD, Wang L, Zhang Y, Phung E, Chang LA, Loomis RJ, Altaras NE, Narayanan E, Metkar M, Presnyak V, Liu C, Louder MK, Shi W, Leung K, Yang ES, West A, Gully KL, Stevens LJ, Wang N, Wrapp D, Doria-Rose NA, Stewart-Jones G, Bennett H, Alvarado GS, Nason MC, Ruckwardt TJ, McLellan JS, Denison MR, Chappell JD, Moore IN, Morabito KM, Mascola JR, Baric RS, Carfi A, Graham BS (2020). SARS-CoV-2 mRNA vaccine design enabled by prototype pathogen preparedness. Nature.

[6] Polack FP, Thomas SJ, Kitchin N, Absalon J, Gurtman A, Lockhart S, Perez JL, Pérez Marc G, Moreira ED, Zerbini C, Bailey R, Swanson KA, Roychoudhury S, Koury K, Li P, Kalina WV, Cooper D, Frenck RW, Hammitt LL, Türeci Ö, Nell H, Schaefer A, Ünal S, Tresnan DB, Mather S, Dormitzer PR, Şahin U, Jansen KU (2020). Gruber, Safety and Efficacy of the BNT162b2 mRNA Covid-19 vaccine, N. Engl. J Med.

[7] Baden LR, El Sahly HM, Essink B, Kotloff K, Frey S, Novak R, Diemert D, Spector SA, Rouphael N, Creech CB, McGettigan J, Khetan S, Segall N, Solis J, Brosz A, Fierro C, Schwartz H, Neuzil K, Corey L, Gilbert P, Janes H, Follmann D, Marovich M, Mascola J, Polakowski L, Ledgerwood J, Graham BS, Bennett H, Pajon R, Knightly C, Leav B, Deng W, Zhou H, Han S, Ivarsson M, Miller J, Zaks T (2021). Efficacy and safety of the mRNA-1273 SARS-CoV-2 Vaccine, N. Engl. J Med.

[8] Szabó GT, Mahiny AJ, Vlatkovic I (2022). COVID-19 mRNA vaccines: platforms and current developments. Mol Ther.

[9] Pardi N, Hogan MJ, Weissman D (2020). Recent advances in mRNA vaccine technology. Curr Opin Immunol.

[10] Alameh M, Weissman D, Pardi N, Messenger RNA-B. Vaccines against infectious diseases. Curr Top Microbiol Immunol. 2020;440. 10.1007/82_2020_202 10.1007/82_2020_202 32300916

[11] Wang Y, Zhang Z, Luo J, Han X, Wei Y, Wei X (2021). mRNA vaccine: a potential therapeutic strategy. Mol Cancer.

[12] Hou X, Zaks T, Langer R, Dong Y (2021). Lipid nanoparticles for mRNA delivery. Nat Rev Mater.

[13] Diallo BK, Ní C, Chasaide TY, Wong P, Schmitt KS, Lee K, Weaver O, Miller M, Cooper SD, Jazayeri FH, Damron KHG, Mills. Intranasal COVID-19 vaccine induces respiratory memory T cells and protects K18-hACE mice against SARS-CoV-2 infection. Npj Vaccines. 2023;8:68. 10.1038/s41541-023-00665-3 10.1038/s41541-023-00665-3 PMC10182552 37179389

[14] Csaba N, Garcia-Fuentes M, Alonso MJ (2009). Nanoparticles for nasal vaccination. Adv Drug Deliv Rev.

[15] Tiboni M, Casettari L, Illum L (2021). Nasal vaccination against SARS-CoV-2: synergistic or alternative to intramuscular vaccines?. Int J Pharm.

[16] Csaba N, Sánchez A, Alonso MJ (2006). Poloxamer and PLGA: poloxamine blend nanostructures as carriers for nasal gene delivery. J Control Release.

[17] Vicente S, Peleteiro M, Díaz-Freitas B, Sanchez A, González-Fernández Á, Alonso MJ (2013). Co-delivery of viral proteins and a TLR7 agonist from polysaccharide nanocapsules: a needle-free vaccination strategy. J Control Release.

[18] Dacoba TG, Omange RW, Li H, Crecente-Campo J, Luo M, Alonso MJ (2019). Polysaccharide nanoparticles can efficiently modulate the Immune response against an HIV peptide Antigen. ACS Nano.

[19] Su X, Fricke J, Kavanagh DG, Irvine DJ (2011). Vitro and in vivo mRNA delivery using lipid-enveloped pH-Responsive polymer nanoparticles. Mol Pharm.

[20] Li M, Zhao M, Fu Y, Li Y, Gong T, Zhang Z, Sun X (2016). Enhanced intranasal delivery of mRNA vaccine by overcoming the nasal epithelial barrier via intra- and paracellular pathways. J Control Release.

[21] Baldeon Vaca G, Meyer M, Cadete A, Hsiao CJ, Golding A, Jeon A, Jacquinet E, Azcue E, Guan CM, Sanchez-Felix X, Pietzsch CA, Mire CE, Hyde MA, Comeaux ME, Williams JM, Sung JC, Carfi A, Edwards DK, Bukreyev A, Bahl K. Intranasal mRNA-LNP vaccination protects hamsters from SARS-CoV-2 infection. Sci Adv. 2023;9. 10.1126/sciadv.adh1655 10.1126/sciadv.adh1655 PMC10516494 37738334

[22] Csaba N, Köping-Höggård M, Alonso MJ (2009). Ionically crosslinked chitosan/tripolyphosphate nanoparticles for oligonucleotide and plasmid DNA delivery. Int J Pharm.

[23] De La Fuente M, Raviña M, Sousa-Herves A, Correa J, Riguera R, Fernandez-Megia E, Sánchez A, Alonso MJ (2012). Exploring the efficiency of gallic acid-based dendrimers and their block copolymers with PEG as gene carriers. Nanomedicine.

[24] Crecente-Campo J, Lorenzo-Abalde S, Mora A, Marzoa J, Csaba N, Blanco J, González-Fernández Á, Alonso MJ (2018). Bilayer polymeric nanocapsules: a formulation approach for a thermostable and adjuvanted E. Coli antigen vaccine. J Control Release.

[25] Cordeiro AS, Crecente-Campo J, Bouzo BL, González SF, de la Fuente M, Alonso MJ (2019). Engineering polymeric nanocapsules for an efficient drainage and biodistribution in the lymphatic system. J Drug Target.

[26] Hoffmann M, Kleine-Weber H, Schroeder S, Krüger N, Herrler T, Erichsen S, Schiergens TS, Herrler G, Wu NH, Nitsche A, Müller MA, Drosten C, Pöhlmann S (2020). SARS-CoV-2 cell entry depends on ACE2 and TMPRSS2 and is blocked by a clinically proven protease inhibitor. Cell.

[27] Marcos-Villar L, Perdiguero B, Anthiya S, Borrajo ML, Lou G, Franceschini L, Esteban I, Sánchez-Cordón PJ, Zamora C, Sorzano CÓS, Jordá L, Codó L, Gelpí JL, Sisteré-Oró M, Meyerhans A, Thielemans K, Martínez-Jiménez F, López-Vigas N, García F, Alonso MJ, Plana M, Esteban M, Gómez CE (2024). Modulating the immune response to SARS-CoV-2 by different nanocarriers delivering an mRNA expressing trimeric RBD of the spike protein: COVARNA Consortium. Npj Vaccines.

[28] Calvo P, Remuñán-López C, Vila-Jato JL, Alonso MJ (1997). Development of positively charged colloidal drug carriers: Chitosan-coated polyester nanocapsules and submicron-emulsions. Colloid Polym Sci.

[29] Ivanov DP, Grabowska AM, Garnett MC (2017). High-throughput spheroid screens using volume, resazurin reduction, and acid phosphatase activity. Methods Mol Biol.

[30] Sharma S, Mukkur TK, Benson HA, Chen Y (2012). Enhanced immune response against pertussis toxoid by IgA-loaded chitosan-dextran sulfate nanoparticles. J Pharm Sci.

[31] Sharma S, Benson HAE, Mukkur TKS, Rigby P, Chen Y (2013). Preliminary studies on the development of IgA-loaded chitosan-dextran sulphate nanoparticles as a potential nasal delivery system for protein antigens. J Microencapsul.

[32] González-Aramundiz JV, Peleteiro Olmedo M, González-Fernández Á, Alonso Fernández MJ, Csaba NS (2015). Protamine-based nanoparticles as new antigen delivery systems. Eur J Pharm Biopharm.

[33] Ruseska I, Fresacher K, Petschacher C, Zimmer A. Use of protamine in nanopharmaceuticals—a review. Nanomaterials. 2021;11. 10.3390/nano11061508 10.3390/nano11061508 PMC8230241 34200384

[34] Lin Y, Sun B, Jin Z, Zhao K (2022). Enhanced Immune responses to Mucosa by Functionalized Chitosan-based Composite nanoparticles as a vaccine adjuvant for Intranasal Delivery. ACS Appl Mater Interfaces.

[35] Streck S, Neumann H, Nielsen HM, Rades T, McDowell A (2019). Comparison of bulk and microfluidics methods for the formulation of poly-lactic-co-glycolic acid (PLGA) nanoparticles modified with cell-penetrating peptides of different architectures. Int J Pharm X.

[36] Fathordoobady F, Sannikova N, Guo Y, Singh A, Kitts DD, Pratap-Singh A (2021). Comparing microfluidics and ultrasonication as formulation methods for developing hempseed oil nanoemulsions for oral delivery applications. Sci Rep.

[37] Gilbert J, Sebastiani F, Arteta MY, Terry A, Fornell A, Russell R, Mahmoudi N, Nylander T (2024). Evolution of the structure of lipid nanoparticles for nucleic acid delivery: from in situ studies of formulation to colloidal stability. J Colloid Interface Sci.

[38] Zhang NN, Li XF, Deng YQ, Zhao H, Huang YJ, Yang G, Huang WJ, Gao P, Zhou C, Zhang RR, Guo Y, Sun SH, Fan H, Zu SL, Chen Q, He Q, Cao TS, Huang XY, Qiu HY, Nie JH, Jiang Y, Yan HY, Ye Q, Zhong X, Xue XL, Zha ZY, Zhou D, Yang X, Wang YC, Ying B, Qin CF (2020). A thermostable mRNA vaccine against COVID-19. Cell.

[39] Bogaert B, Sauvage F, Guagliardo R, Muntean C, Nguyen VP, Pottie E, Wels M, Minnaert AK, De Rycke R, Yang Q, Peer D, Sanders N, Remaut K, Paulus YM, Stove C, De Smedt SC, Raemdonck K (2022). A lipid nanoparticle platform for mRNA delivery through repurposing of cationic amphiphilic drugs. J Control Release.

[40] Shao X, Wei X, Song X, Hao L, Cai X, Zhang Z, Peng Q, Lin Y (2015). Independent effect of polymeric nanoparticle zeta potential/surface charge, on their cytotoxicity and affinity to cells. Cell Prolif.

[41] Bhattacharjee S, de Haan LH, Evers NM, Jiang X, Marcelis AT, Zuilhof H, Rietjens IM, Alink GM (2010). Role of surface charge and oxidative stress in cytotoxicity of organic monolayer-coated silicon nanoparticles towards macrophage NR8383 cells, part. Fibre Toxicol.

[42] You J, Kamihira M, Iijima S (1997). Surfactant-mediated gene transfer for animal cells. Cytotechnology.

[43] Kim B-K, Hwang G-B, Seu Y-B, Choi J-S, Jin KS, Doh K-O (2015). DOTAP/DOPE ratio and cell type determine transfection efficiency with DOTAP-liposomes, Biochim. Biophys Acta - Biomembr.

[44] Blakney AK, McKay PF, Yus BI, Aldon Y, Shattock RJ (2019). Inside out: optimization of lipid nanoparticle formulations for exterior complexation and in vivo delivery of saRNA. Gene Ther.

[45] Han X, Zhang H, Butowska K, Swingle KL, Alameh MG, Weissman D, Mitchell MJ (2021). An ionizable lipid toolbox for RNA delivery. Nat Commun.

[46] Carrasco MJ, Alishetty S, Alameh M-G, Said H, Wright L, Paige M, Soliman O, Weissman D, Cleveland TE, Grishaev A, Buschmann MD (2021). Ionization and structural properties of mRNA lipid nanoparticles influence expression in intramuscular and intravascular administration. Commun Biol.

[47] Di J, Du Z, Wu K, Jin S, Wang X, Li T, Xu Y (2022). Biodistribution and non-linear gene expression of mRNA LNPs affected by Delivery Route and particle size. Pharm Res.

[48] Semple SC, Akinc A, Chen J, Sandhu AP, Mui BL, Cho CK, Sah DWY, Stebbing D, Crosley EJ, Yaworski E, Hafez IM, Dorkin JR, Qin J, Lam K, Rajeev KG, Wong KF, Jeffs LB, Nechev L, Eisenhardt ML, Jayaraman M, Kazem M, Maier MA, Srinivasulu M, Weinstein MJ, Chen Q, Alvarez R, Barros SA, De S, Klimuk SK, Borland T, Kosovrasti V, Cantley WL, Tam YK, Manoharan M, Ciufolini MA, Tracy MA, De Fougerolles A, MacLachlan I, Cullis PR, Madden TD, Hope MJ (2010). Rational design of cationic lipids for siRNA delivery. Nat Biotechnol.

[49] Garulli B, Stillitano MG, Barnaba V, Castrucci MR (2008). Primary CD8 + T-cell response to soluble ovalbumin is improved by chloroquine treatment in vivo. Clin Vaccine Immunol.

[50] Dacoba TG, Olivera A, Torres D, Crecente-Campo J, Alonso MJ (2017). Modulating the immune system through nanotechnology. Semin Immunol.

[51] Moss P (2022). The T cell immune response against SARS-CoV-2. Nat Immunol.

[52] Mai Y, Guo J, Zhao Y, Ma S, Hou Y, Yang J (2020). Intranasal delivery of cationic liposome-protamine complex mRNA vaccine elicits effective anti-tumor immunity. Cell Immunol.

[53] Phua KKL, Staats HF, Leong KW, Nair SK (2014). Intranasal mRNA nanoparticle vaccination induces prophylactic and therapeutic anti-tumor immunity. Sci Rep.

[54] Tario JD, Chen GL, Hahn TE, Pan D, Furlage RL, Zhang Y, Brix L, Halgreen C, Jacobsen K, McCarthy PL, Wallace PK (2015). Dextramer reagents are effective tools for quantifying CMV antigen-specific T cells from peripheral blood samples, Cytom. Part B Clin Cytom.

