# Supplementary material for: Nanoemulsions and nanocapsules as carriers for the development of intranasal mRNA vaccines
Source: Drug Deliv Transl Res. 2024 May 29;14(8):2046–61. doi: 10.1007/s13346-024-01635-5 (PMC11208213; doi:10.1007/s13346-024-01635-5)
Supplement: Supplementary file 1 — Supplementary Material 1 [file 13346_2024_1635_MOESM1_ESM.docx]

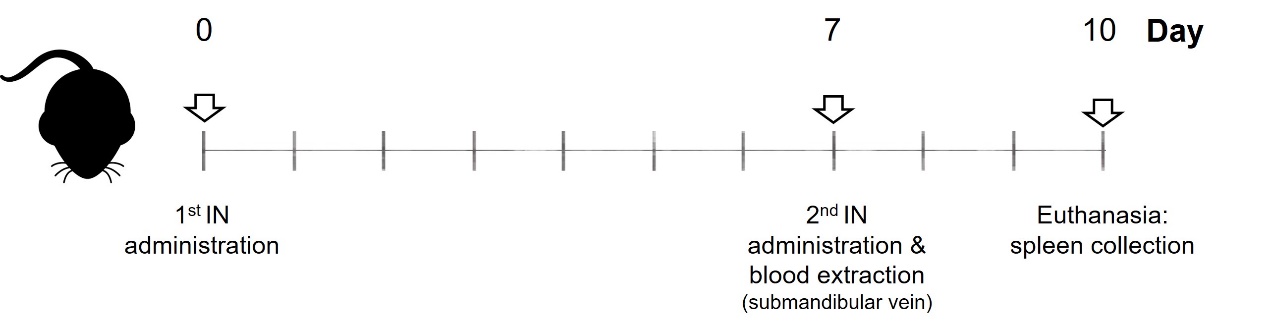


**Supplementary Fig. 1. Intranasal administration protocol for NE-13-mOVA and NC-4-DX-mOVA. Administration was performed at day 0 and 7 in C57BL/6J female mice (6–8 weeks old). Blood samples from submandibular vein were collected on day 7, and mice were euthanized on day 10, followed by spleen collection.**

**Abbreviations:** DX: dextran sulphate. IN: intranasal. mOVA: mRNA encoding for ovalbumin. NE: nanoemulsion. NC: nanocapsule.
