# Supplementary material for: Nanoemulsions and nanocapsules as carriers for the development of intranasal mRNA vaccines
Source: Drug Deliv Transl Res. 2024 May 29;14(8):2046–61. doi: 10.1007/s13346-024-01635-5 (PMC11208213; doi:10.1007/s13346-024-01635-5)
Supplement: Supplementary file 2 — Supplementary Material 2 [file 13346_2024_1635_MOESM2_ESM.docx]

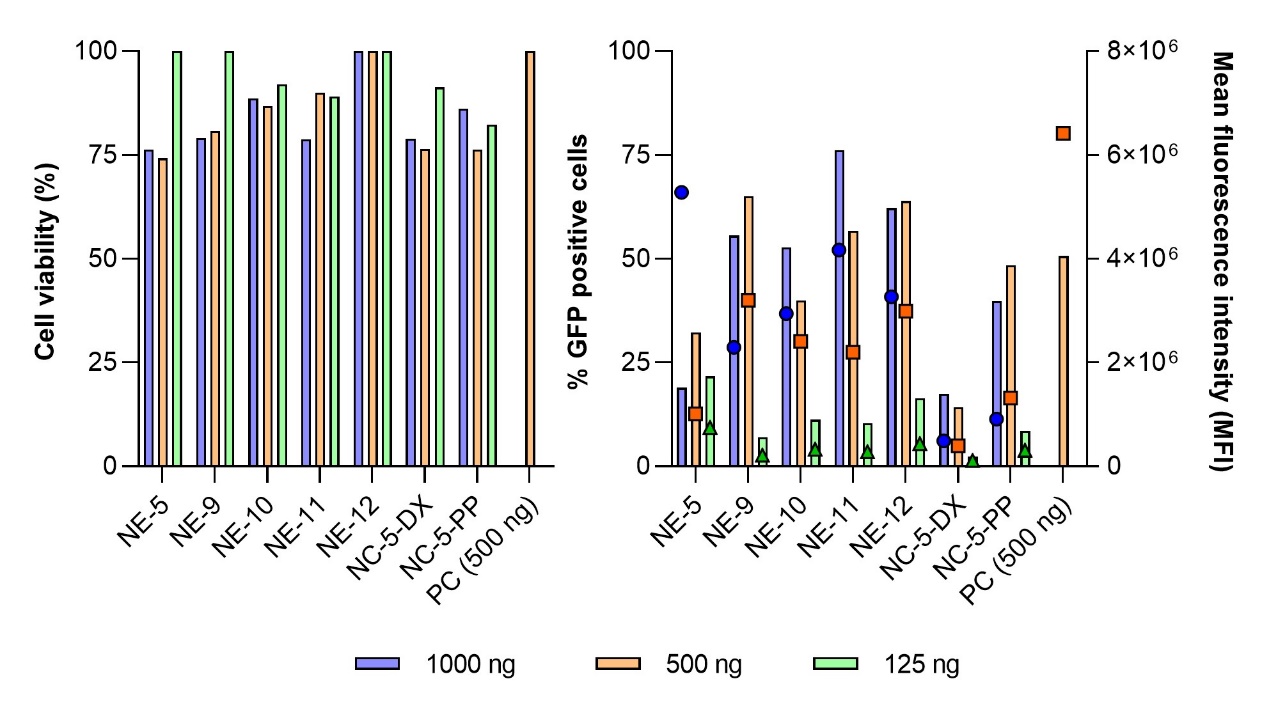


**Supplementary Fig. 2. Cytotoxicity (left) and GFP transfection (right) of NE-3-mGFP, NE-9-mGFP, NE-10-mGFP, NE-11-mGFP, NE-12-mGFP, NC-5-DX-mGFP and NC-5-PP-mGFP, at different mGFP concentrations. Transfection efficiency is expressed in percentage of GFP positive cells (left axis) and mean fluorescence intensity (right axis) in HeLa cells, 24 hours post transfection.**

**Abbreviations:** DX: dextran sulphate. mGFP: mRNA encoding for GFP. NE: nanoemulsion. NC: nanocapsule. PC: positive control, lipofectamine. PP: PGA-PEG or PEG (5 kDa)-b-PGA (10) (Na). (n = 1).
