# Supplementary material for: Nanoemulsions and nanocapsules as carriers for the development of intranasal mRNA vaccines
Source: Drug Deliv Transl Res. 2024 May 29;14(8):2046–61. doi: 10.1007/s13346-024-01635-5 (PMC11208213; doi:10.1007/s13346-024-01635-5)
Supplement: Supplementary file 3 — Supplementary Material 3 [file 13346_2024_1635_MOESM3_ESM.docx]

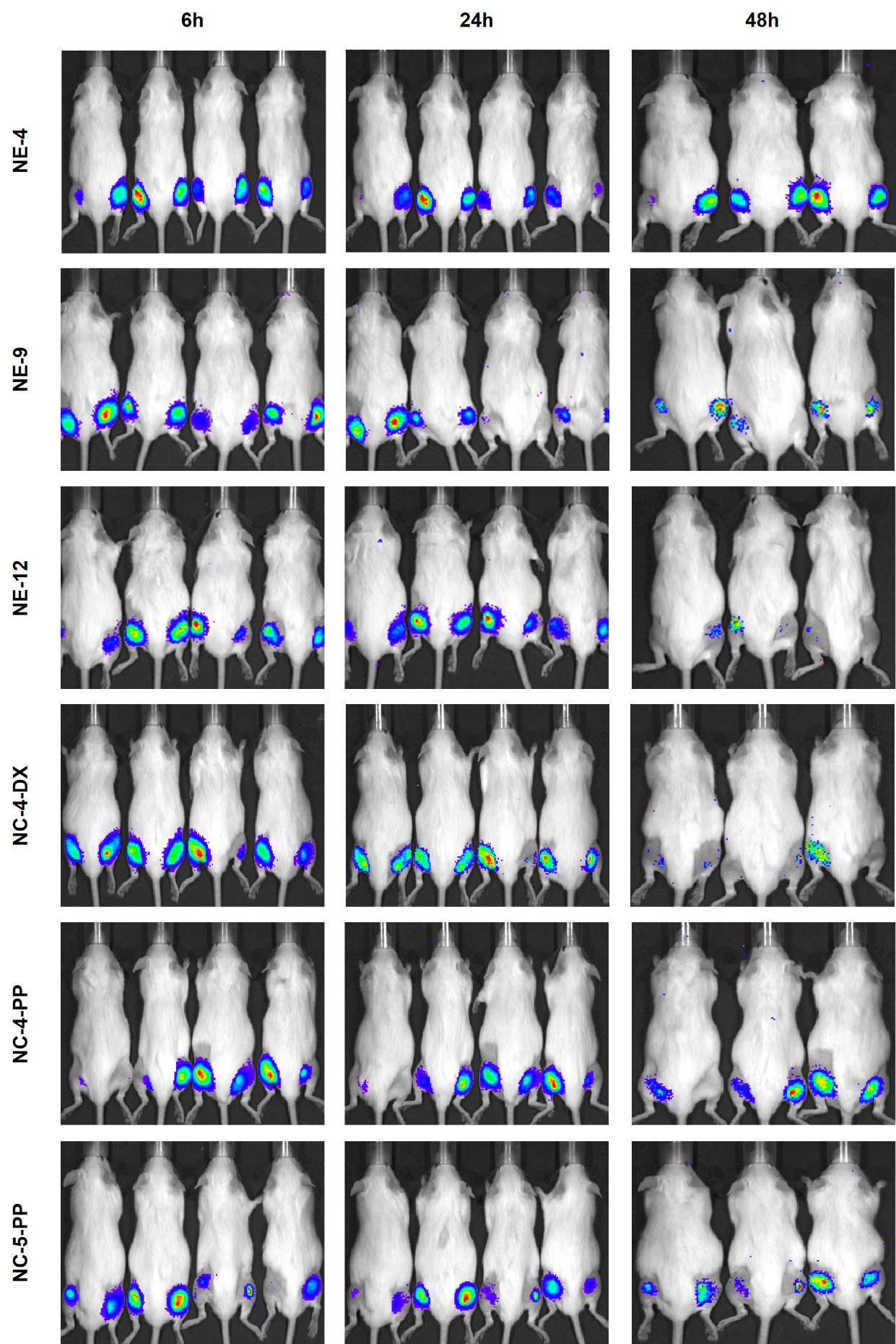


**Supplementary Fig. 3. Luciferase fluorescence signal detected by IVIS using NE-4-mLuc, NE-9-mLuc, NE-12-mLuc, NC-4-DX-mLuc, NC-4-PP-mLuc, and NC-5-PP-mLuc at different time points (6,24, and 48 hours) after intramuscular administration.**

**Abbreviations:** DX: dextran sulphate. mLuc: mRNA encoding for luciferase. NE: nanoemulsion. NC: nanocapsule. PP: PGA-PEG or PEG (5 kDa)-b-PGA (10) (Na).
