# Supplementary material for: Nanoemulsions and nanocapsules as carriers for the development of intranasal mRNA vaccines
Source: Drug Deliv Transl Res. 2024 May 29;14(8):2046–61. doi: 10.1007/s13346-024-01635-5 (PMC11208213; doi:10.1007/s13346-024-01635-5)
Supplement: Supplementary file 4 — Supplementary Material 4 [file 13346_2024_1635_MOESM4_ESM.docx]

**Supplementary Table 1. Physicochemical properties of selected NE-mRNAs and NC-mRNAs before and after lyophilization process in present of different cryoprotectants.**

| Code | Type of mRNA | Condition | Size (nm) | PDI | Z-Pot (mV) |
| --- | --- | --- | --- | --- | --- |
| NE-3 | mGFP (n = 1) | Before lyophilization | 93 | 0.12 | + 36 |
|  |  | Sucrose 10% | 122 | 0.20 | + 34 |
|  | mLuc | Before lyophilization | 112 ± 16 | 0.17 ± 0.01 | + 35 ± 6 |
|  |  | Sucrose 20% (n = 1) | 100 | 0.18 | + 35 |
| NE-4 | mGFP (n = 1) | Before lyophilization | 128 | 0.11 | + 42 |
|  |  | Sucrose 10% | 168 | 0.17 | + 42 |
|  | mLuc (n = 1) | Before lyophilization | 123 | 0.22 | + 45 |
|  |  | Sucrose 20% | 116 | 0.14 | + 44 |
| NE-9 | mLuc | Before lyophilization | 100 ± 3 | 0.17 ± 0.02 | + 56 ± 7 |
|  |  | Sucrose 20% | 119 ± 14 | 0.15 ± 0.02 | + 47 ± 4 |
| NC-3-PR | mGFP (n = 1) | Before lyophilization | 154 | 0.11 | + 28 |
|  |  | Trehalose 10% | 174 | 0.17 | + 29 |
|  | mLuc (n = 1) | Before lyophilization | 179 | 0.16 | + 24 |
|  |  | Trehalose 10% | 180 | 0.16 | + 24 |
| NC-4-PR | mGFP (n = 1) | Before lyophilization | 168 | 0.10 | + 27 |
|  |  | Trehalose 10% | 185 | 0.07 | + 31 |
| NC-4-DX | mLuc | Before lyophilization | 139 ± 21 | 0.14 ± 0.05 | -19 ± 4 |
|  |  | Sucrose 10% | 162 ± 20 | 0.12 ± 0.07 | -30 ± 11 |

**Abbreviations:** DX: dextran sulphate. mGFP: mRNA encoding for GFP. mLuc: mRNA encoding for luciferase. NC: nanocapsule. NE: nanoemulsion. PDI: polydispersity index. PR: protamine sulphate EP. Values represent the mean ± standard deviation (n ≥ 3, unless indicated otherwise).
